# Supplementary material for: Theoretical principles of transcription factor traffic on folded chromatin
Source: Nat Commun. 2018 Apr 30;9:1740. doi: 10.1038/s41467-018-04130-x (PMC5928121; doi:10.1038/s41467-018-04130-x)
Supplement: Supplementary file 1 — Supplementary Information [file 41467_2018_4130_MOESM1_ESM.pdf]

Supplementary Information for

Theoretical principles of transcription factor traffic on folded chromatin

Ruggero Cortini[1,2*] and Guillaume J. Filion[1,2*]

[1] *Genome Architecture, Gene Regulation, Stem Cells and Cancer Programme, Centre for Genomic Regulation (CRG), The Barcelona Institute of Science and Technology, Dr. Aiguader 88, Barcelona 08003, Spain*

[2] *Universidad Pompeu Fabra (UPF), Barcelona 08003, Spain*

[*] *Correspondence to: ruggero.cortini@crg.eu*

[*] *Correspondence to: guillaume.filion@gmail.com*

April 3, 2018

This PDF file includes:

- Supplementary Notes 1 to 7
- Supplementary Figures 1 to 7
- Supplementary References

Supplementary Note 1: Simulations with 200 tracers

The results presented in the Main Text referred to simulations performed with 10 tracers. The small number of tracers ensures that they cause minimal perturbations to the polymer structure. On the other hand, it is interesting to ask in which conditions would the tracers themselves perturb significantly the conformation of the polymer. We therefore performed a large simulation round in the same setup as the one described in the Main Text for 10 tracers, but with 200 tracers. In this section we illustrate the main effects of the increased number of tracers on the traffic of the tracers.

Supplementary Fig. 1 shows the average values of the KL divergence (a), the Pearson correlation between tracer traffic and polymer contacts (b), the coverage (c) and the Pearson correlation between tracer traffic and binder traffic (d). By comparing the results from this simulation round to the ones obtained by simulating 10 tracers, we see that the main conclusions stay the same. In the next Supplementary Note we show the evidence that the polymer conformation changes due to the contacts created by the tracers in the case of 200 tracers.

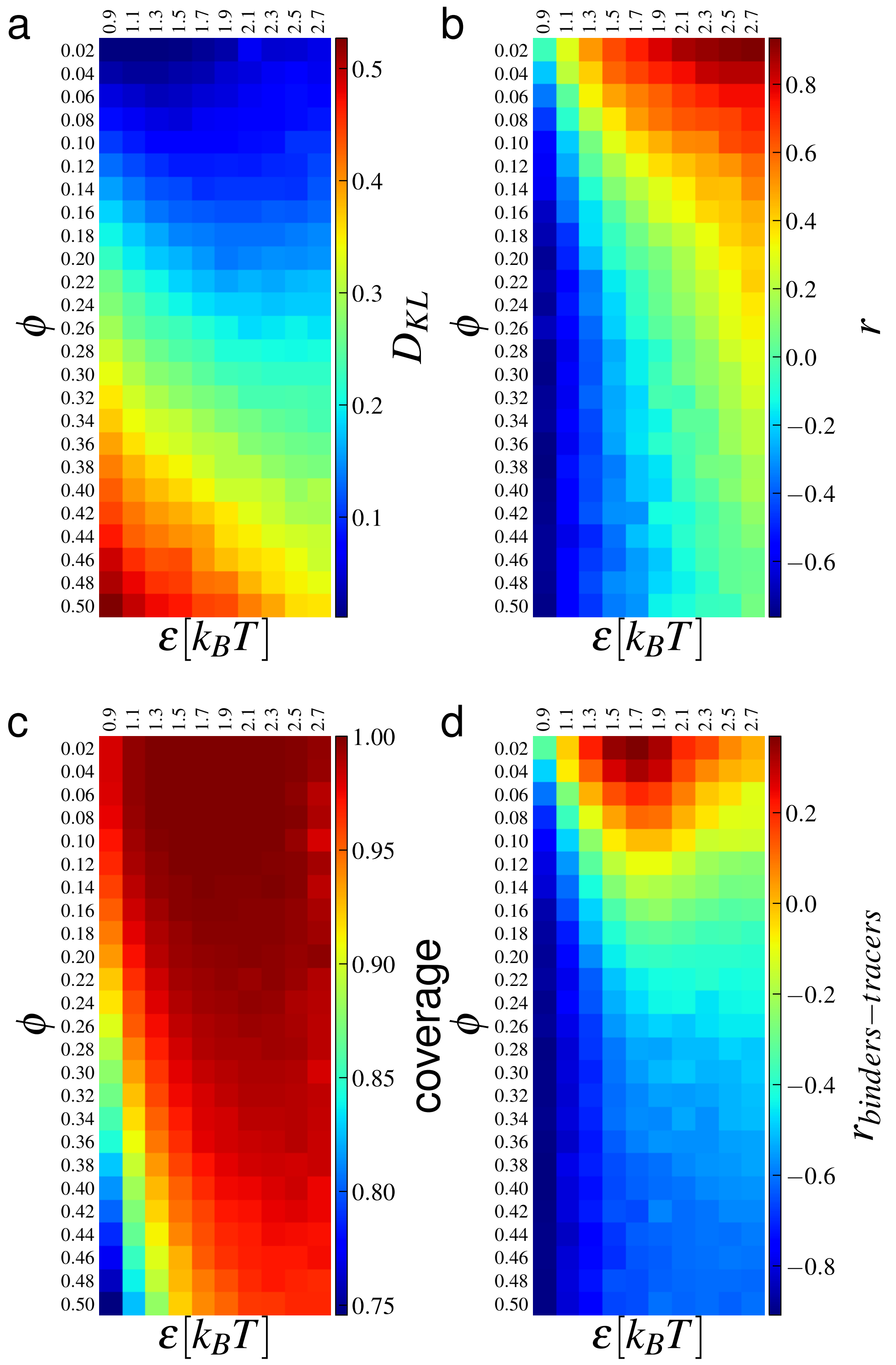

Supplementary Figure 1: Results of the simulations with 200 tracers. Average Kullback-Leibler divergence (a) and Pearson correlation coefficient (b) between the tracer traffic and the polymer contacts. (c) Average coverage of the tracers. (d) Average Pearson correlation coefficient between the contacts made by the tracers (traffic) and the contacts made by the binders.

Supplementary Note 2: Polymer conformation

We investigated the properties of the polymer configuration in our simulation sets. First, we looked at the radius of gyration of the polymer, which is defined as follows:

$$r_{gyr}^2 = \frac{1}{N} \sum_{i=1}^{N} \left\langle (\mathbf{r}_i - \mathbf{r_{cm}})^2 \right\rangle , \tag{1}$$

where $\mathbf{r}_{cm}$ is the position vector of the center of mass of the polymer. Supplementary Fig. 2a and 2b show the average values of the radius of gyration, calculated by averaging over the last $5 \cdot 10^7$ trajectory frames of the simulations. In the case of $n_t = 10$, the radius of gyration remains roughly constant for all values of ε. However, when $n_t = 200$ there is a sharp decrease of the radius of gyration as ε increases. This is a sign that the tracers are driving a coil-globule transition in the polymer and that the tracers are themselves creating the contacts between distal polymer segments.

To further demonstrate this, we examined $p(s)$, which represents the average probability of contacts between monomers separated by $s - 1$ other monomers. From classical polymer physics arguments, we expect that $p(s) \sim s^{-\alpha}$, where α is an exponent that depends on the polymer interactions and on the particular state of the polymer. Supplementary Fig. 2c and 2d show the average values of $p(s)$ depicted for a representative sample of values of ϕ and for $n_t = 10$ or $n_t = 200$. For a given value of ϕ, we can define the average minimal loop size l as the average separation between binding sites (in number of monomers), that is $l = N\phi$. Up to $s \approx l$ the $p(s)$ function is dominated by the contribution of minimal loops. It was shown [1] that up to half of this distance $\alpha \approx 2.20$. The decay observed in our simulations is in good agreement with this expectation. Comparing Supplementary Figs. 2c and 2d further show that for $s > l$, the decay rate α is substantially lower in the simulations with 200 tracers, showing that the polymer is more compact in this case.

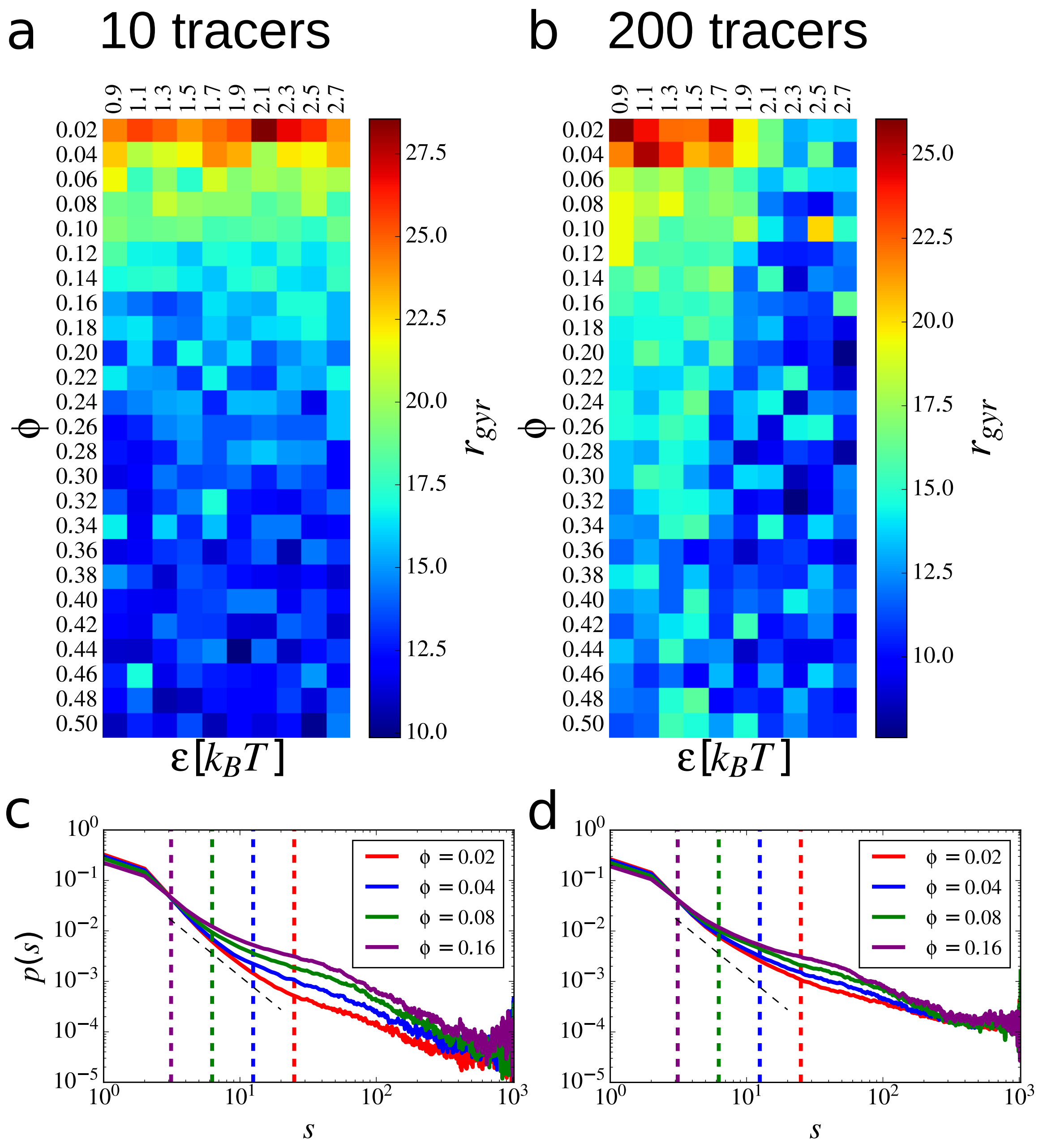

Supplementary Figure 2: Polymer conformation. (a,b) Average radius of gyration as a function of the two parameters ϕ and ε. (c,d) Average probability of contact $p(s)$ as a function of the linear distance along the polymer s. Curves are shown for four different values of ϕ. The vertical dashed lines correspond to the length of the shortest half-loop l at the given value of ϕ. Theory predicts that the decay rate α is approximately 2.20 for $s < l/2$.

Supplementary Note 3: Diffusion of the tracers

To assess the diffusive properties of the tracers, we calculated the mean square displacement (MSD) of the tracers, as follows:

$$\mathrm{MSD}_i(\Delta t) = \left\langle (\mathbf{r}_i(t_1) - \mathbf{r}_i(t_0))^2 \right\rangle, \qquad \Delta t = t_1 - t_0. \tag{2}$$

Here $\mathbf{r}_i$ is the position vector of the i-th tracer, and the average $\langle \ldots \rangle$ is performed over the statistically independent snapshots. The calculation of the MSD was performed by averaging over many possible independent starting times, following the guidelines in Ref. [2]. For sufficiently long time intervals, the values of the MSD as a function of time obey the relationship

$$\mathrm{MSD}_i(\Delta t) = 6Dt \tag{3}$$

where D is the diffusion coefficient. For a tracer that does not have any interactions with other particles in the system, we expect that the value of the diffusion coefficient should be given by the Einstein relationship:

$$D_0 = \frac{k_B T}{\gamma}, \tag{4}$$

where γ is the drag coefficient of the particle.

In general, however, it is interesting to look at the instantaneous diffusion coefficient, which is obtained from the MSD time trace by taking its time derivative:

$$D_{inst} \equiv \frac{1}{6}\frac{\mathrm{d}}{\mathrm{d}t}\left[\mathrm{MSD}(t)\right]. \tag{5}$$

For each of the diffusing tracers we expect a different value of the instantaneous diffusion coefficient, because each tracer will randomly bind and unbind to the polymer in the simulation. Therefore, the most useful representation of the average properties of the diffusion in each simulation is the plot of the distribution of instantaneous diffusion coefficients, because the parameters in the simulations vary.

We investigated the relationship between the diffusion properties of the tracers and their affinity to the polymer, together with the three-dimensional structure of the polymer. Supplementary Fig. 3 summarizes our finding on the diffusion behavior of the tracers. Supplementary Fig. 3 shows the MSD curves as a function of time for the ten tracers in an example simulation. For the chosen value of $\varepsilon = 2.1 k_B T$, the tracers have markedly different behaviors: some of them have a steep MSD curve with slope close to the value of the theoretically expected diffusion coefficient D_0 (see Supplementary Equation (4)), whereas other tracers are almost immobile (flat MSD(t) curve).

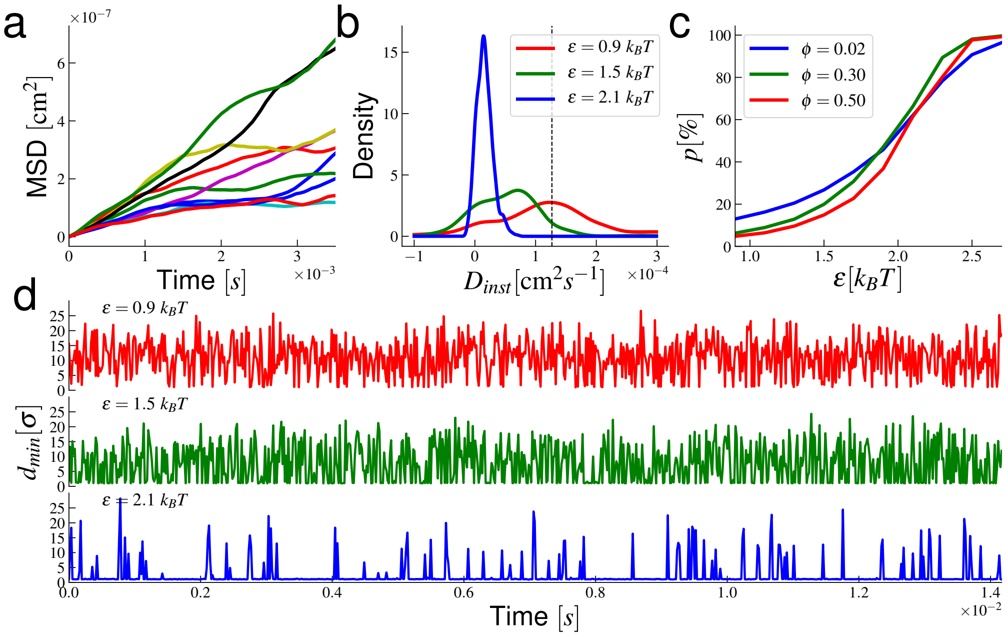

Supplementary Figure 3: Diffusion properties of the tracers in the simulations. (a) MSD as a function of time for the tracers in an example simulation at $\phi = 0.20$, $\varepsilon = 2.1 k_BT$ and $n_t = 10$. (b) Distribution of instantaneous diffusion coefficients for three example simulations at different values of $\varepsilon = 0.9, 1.5, 2.1 k_BT$ and $\phi = 0.20$. The dashed vertical line corresponds to the value of D_0 (see Supplementary Equation (4)). (c) Percentage of binding events p as a function of the tracer-polymer affinity, for three different values of ϕ. The value of p corresponds to the average fraction of simulation frames in which the minimum distance between the tracer and the polymer d_{min} is lower than the Lennard-Jones cutoff length r_{cut}. (d) Three example time traces of d_{min} as a function of the simulation time, for three different values of the tracer-polymer affinity ε.

To characterize further this behavior, we calculated the distribution of instantaneous diffusion coefficients. Supplementary Fig. 3b shows the distribution of D_{inst} for three different simulations, for a fixed value of $\phi = 0.20$. As ε grows, the instantaneous diffusion coefficients become smaller. However, there is always a fraction of the tracers that has instantaneous diffusion coefficient close to D_0 (dashed vertical line in the figure).

We also investigated the effect of the three-dimensional structure of the polymer on the diffusion properties of the tracers. To this end, we calculated the minimum distance between a tracer and the polymer (d_{min}), and the percentage p of frames in the simulations in which d_{min} is smaller than t, the threshold for the definition of a contact. Supplementary Fig. 3c shows the variation of p as a function of ε, for three fixed values of ϕ. In every case, there is a monotonic increase of the percentage of binding events when ε increases. The same can be seen in Supplementary Fig. 3d, where the time traces of three example tracers are plotted, for three different values of ε. The effect of the three-

dimensional structure of the polymer, as proxied by the value of ϕ, is less pronounced than the effect of ε. Supplementary Fig. 3c shows that lower values of ϕ correspond to higher values of p, but only for small tracer-polymer affinity. This is again an indication that high values of ϕ hinder binding of the tracers.

Supplementary Note 4: Monovalent tracers

So far, tracers were modelled as spherical particles that can make any number of contacts with the polymer. From an energetic point of view, this will result in a more stable configuration when making multiple contacts with the polymer. This engenders two potential issues: the first is that the intra-polymer contacts themselves may be created by the tracers; the second is that such promiscuous interactions may not represent realistic transcription factors. The first issue has been addressed in Supplementary Notes 1 and 2, showing that this is indeed the case. To address the second issue and more generally to determine if the main results are crucially dependent on the assumption that tracers are multivalent, we performed another large-scale simulation round using monovalent tracers.

The only difference with the simulation setup described in the Main Text were how the tracers are modelled, and how the molecular dynamics integration is performed. We used the same approach of Brackley *et al.* in modelling monovalent tracers [3]. The monovalent tracers consist of a composite particle (rigid body), made of one spherical particle of diameter $\sigma = 1$ as before (the tracer *per se*), rigidly attached to a smaller spherical particle (which we call m) at a distance $d_m = 0.4\sigma$ from the center of the tracer, and of diameter $\sigma_m = \sigma/2$. Importantly, the m particles are the only particles that have an attractive interaction to the polymer particles, whereas the tracers per se interact with all other particles in the system only through hard-core repulsion. Whenever two particles of different diameters interact, we chose the parameter of the Lennard-Jones interactions (see Equation (3) of the Main Text) as being the standard average between the two interacting particles:

$$\sigma_{ij} = \frac{\sigma_i + \sigma_j}{2}. \tag{6}$$

The tracers were initialized in the system as before, and the position of the m particles was set to a random orientation with respect to the center of the tracers. The integration of the system was performed with the rigid body integrator of HOOMD-blue, which automatically takes care of the integration of rigidly connected particles.

As in the other cases, we performed 10 independent simulations for each pair of values of ϕ and ε. Here, we tested values of ε up to $4.5k_BT$. The interactions are somewhat weaker due to the fact that the interacting portion of the tracer is smaller than for multivalent tracers, which we compensated by increasing the affinity.

The results of this simulation set are illustrated in Supplementary Fig. 4. Supplementary Fig. 4a and 4b show examples of simulation results with high affinity and low compaction, and of low affinity and high compaction (as in Figure 2 of the Main Text). Notice that since the tracers are monovalent, there is a strong depletion of tracer traffic at anchor sites on the polymer: monovalent tracers do not benefit from the energetic

advantage that multivalent particles have. This effect barely changed the value of the Kullback-Leibler divergence, but it strongly decreased the Pearson correlation coefficient between the tracer traffic and the polymer contacts. However, by excluding the anchor sites from the analysis, we quantitatively recovered the results observed with multivalent tracers.

The average values of the KL divergence (with anchor sites) and Pearson correlation coefficient (without anchor sites) are depicted in Supplementary Fig. 4c and 4d. Compared with Figure 4 of the Main Text, it is clear that the qualitative picture that emerges from this simulation assay is the same as the one that emerged with multivalent tracers. This shows that our results are robust with respect to the details of how we treat the tracers in the system regarding their valency.

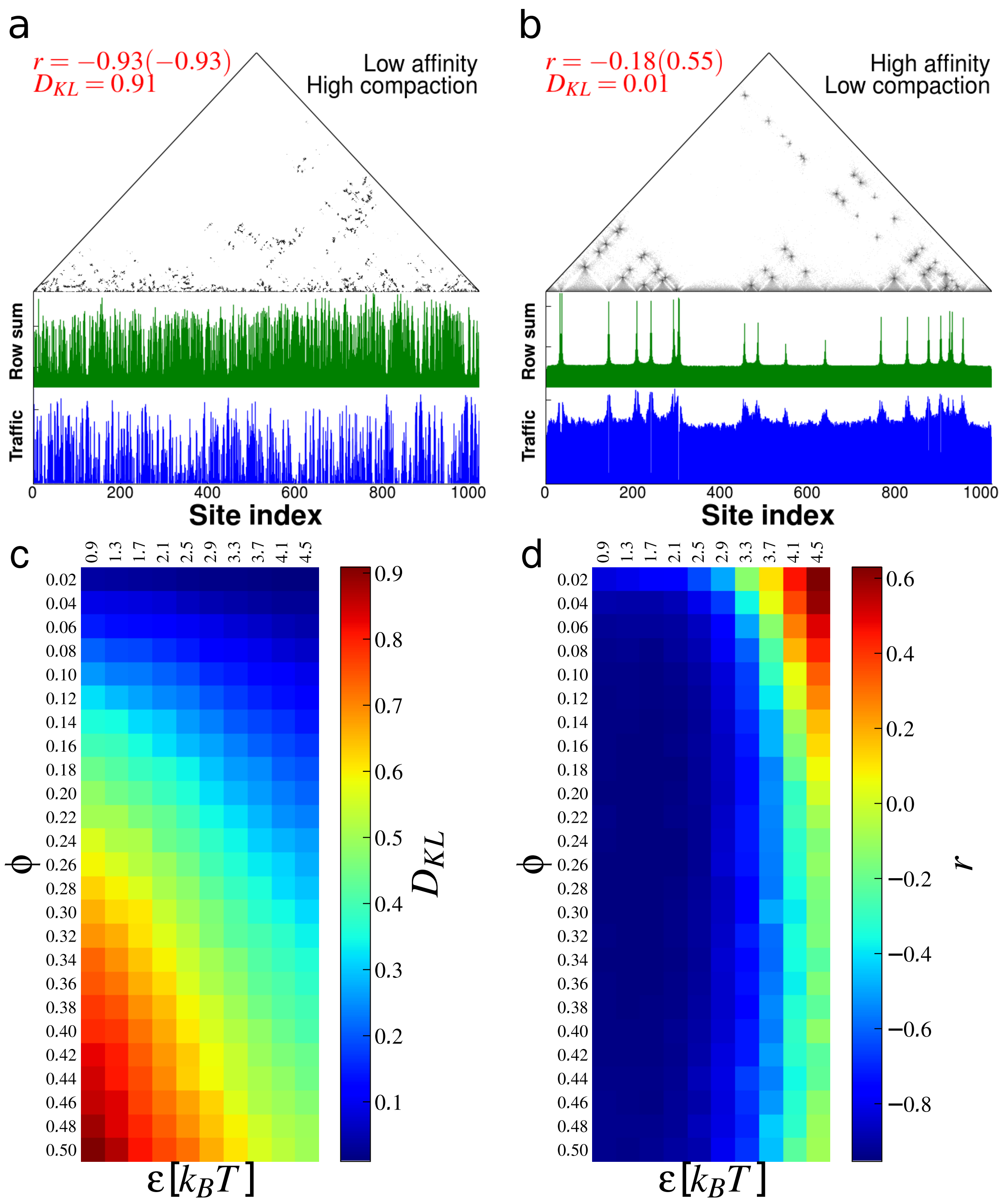

Supplementary Figure 4: Results of monovalent tracer simulations. (a,b) Examples of simulation results. In the upper left corner we report the value of the Pearson correlation between the intra-polymer contact matrix row sum and the traffic of the tracers, along with the Kullback-Leibler divergence. In parentheses we show the value of the Pearson correlation when not taking into account the anchor sites on the polymer (see text for further details). (c) Average values of the Kullback-Leibler divergence and (d) average values of the Pearson correlation coefficient without taking into account anchor sites.

Supplementary Note 5: Dependence on the tracer diameter

The main claims of our paper are relationships between the large-scale structure of a polymer and the traffic of particles that have an affinity for it. Intuitively, such effects are dependent on some ratio between the characteristic size of the particles trafficking on the polymer, and the scale of the polymer itself. For this reason, we sought to investigate how our results are affected by the size (diameter) of the tracers, which we dubbed σ_t.

We varied the diameter of the tracers from sub-unity, i.e., smaller than the diameter of the particle that compose the polymer, to 3 times larger than this diameter. To see how this affected our main conclusions, we performed the simulations in the four corners of the parameter space, where the $(\phi, \varepsilon[k_B T])$ pairs take respective values (0.02,0.9), (0.02,2.7), (0.50, 0.9) and (0.50, 2.7).

We ran two independent simulation sets. In the first, the tracers were treated as before, and as such can make any number of contacts with the polymer. When tracers of this type are larger than the characteristic size of the polymer, and the tracer affinity is high enough, the tracer themselves create a significant amount of contacts (see Supplementary Fig. 5a and 5b). To avoid this potentially artificial situation, we also ran a second simulation set with monovalent tracers where $\sigma_t > 1$. The simulation setup for monovalent tracers is the same as the one described in Supplementary Note 4.

The main results of this round of simulation are shown in Supplementary Fig. 5e-h. Here, we show the values of the Pearson correlation coefficient between the row sum of the intra-polymer contact matrix and the traffic of the tracers, both in the case of monovalent and multivalent tracer simulations. As observed in Supplementary Note 4, for the same values of all the parameters, the Pearson correlation coefficient r differs for monovalent and multivalent tracers. Note that the effect of varying the tracer diameter is most pronounced for multivalent tracers. In this case and as mentioned earlier, a larger tracer with a high affinity to the polymer will strongly perturb the polymer structure, as visually shown in Supplementary Fig. 5a.

In the case of low compaction, multivalent and monovalent tracers have a distinct behaviour when increasing the tracer diameter, both at low and high affinities. Multivalent tracers can penetrate easily in the structure of the polymer, thereby creating the polymer contacts: clearly the polymer contacts will be correlated to the tracer contacts in this case. However, monovalent tracers do not perturb so heavily the structure of the polymer. Instead, they are excluded from the sites where the polymer makes loops (Supplementary Fig. 4b). Therefore, as the tracer diameter increases, the Pearson correlation coefficient becomes more and more negative.

The case of high compaction is more complex. For monovalent tracers, increasing the tracer diameter has no effect, as the tracers have a characteristic size that is larger than

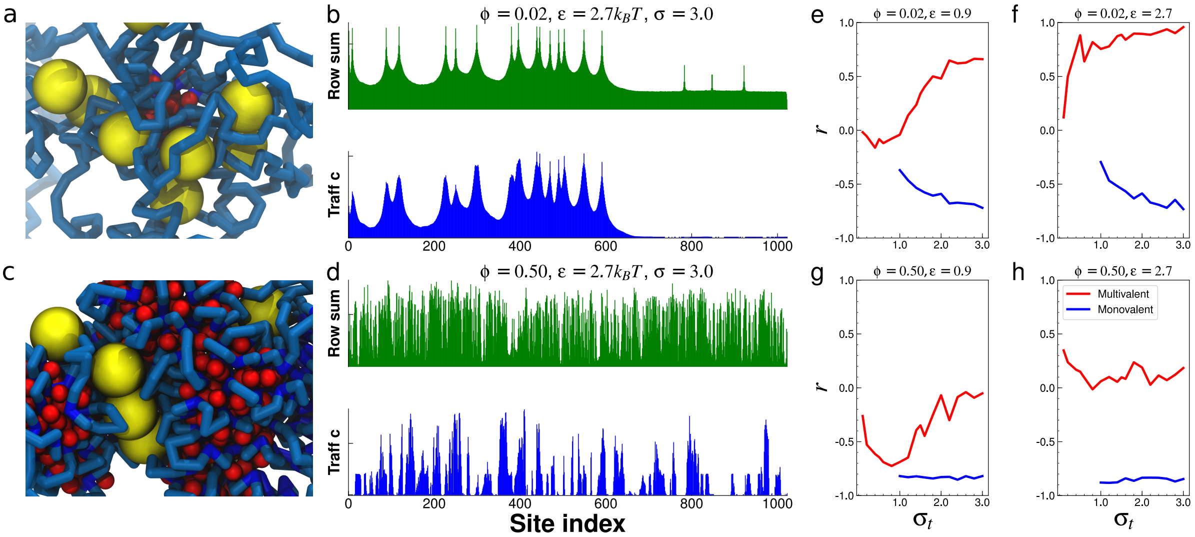

Supplementary Figure 5: Effects of varying tracer diameter. (a) Snapshot of a simulation with low compaction, high affinity and high multivalent tracer diameter along with its polymer contacts and traffic (b). (c) Snapshot of a simulation with high compaction, high affinity and high multivalent tracer diameter, along with its polymer contacts and traffic (d). (e-h) Pearson correlation coefficient between the polymer contacts and tracer traffic in the four corners of the explored parameter space. In red, simulations with multivalent tracers, in blue, simulations with monovalent tracers at the same value of ε.

the "pores" in the polymer, and do not penetrate the polymer core. As a result, the Pearson correlation coefficient is negative and constant as σ_t increases.

For multivalent tracers at high compaction, the behaviour is different at low and high affinity. At low affinity, r is negative throughout the explored values of σ_t, but interestingly, it has a minimum at $\sigma_t \approx 1$. This is due to the fact that at the volume exclusion effect that we discussed in the Main Text is at its maximum efficacy, and the polymer excludes the tracers. For $\sigma_t < 1$, the polymer contacts and the tracer traffic are mostly unrelated, so $r \approx 0$. When $\sigma_t > 1$, the large tracers may bind strongly both to sites of low polymer contacts and get trapped as in a cage, or accumulate at sites of multiple polymer contacts, benefiting from an energetic advantage. Therefore the pattern of tracer traffic becomes unrelated to the polymer contacts, and again $r \approx 0$. If the tracer affinity is high, on the other hand, then we observe a modest positive correlation between the polymer contacts and the tracer traffic at all values of σ_t. That is because the high tracer affinity strongly affects the polymer conformation, and results in an effect similar to the one discussed earlier for low compaction (Supplementary Fig. 4b-d).

To summarize, varying the diameter of the tracers has mixed effects that depend on the details of the polymer structure and the interactions between the tracers and the polymer. We conclude that the theory developed here holds for particles of size comparable to the three-dimensional structures made by the polymer. The cases of particles that are much

smaller or much larger are worth exploring in more detail in forthcoming work.

Supplementary Note 6: Crowding

In the results presented in the Main Text and in the other Supplementary Notes, the number of particles present in the simulation amounts to a total volume that covers about 1% of the simulation box. The most recent estimates of the global volume occupancy inside a cell nucleus go from 20 to 40 %[4]. Therefore, an interesting question is whether the results of our simulations are robust with respect to crowding, that is, at volume fractions that are more relevant physiologically.

To test this, we used the simulation setup presented in the Main Text, except that we added a certain number, n_c of crowding particles with diameter σ_c (in local units) to obtain a final total volume occupancy equal to ρ. It is easy to show that the number of crowders to add is equal to

$$n_c = \frac{6L^3\rho}{\pi\sigma_c^3} - N(1+2\phi)\left(\frac{\sigma}{\sigma_c}\right)^3 \tag{7}$$

where L is the edge length of the simulation box, N is the number of monomers in the polymer, and $\sigma = 1$ is the diameter of the monomers and binders. In this equation we neglected the contribution of the tracers to the total volume because the number of tracers is very small compared to the number of other particles. The crowders interact with all other particles through a purely repulsive Lennard-Jones potential (see Equation (3) of the Main Text) setting $\xi_{ij} = 0$, and an interaction radius that corresponds to Supplementary Equation (6). We simulated the system with total volume fraction going from 5% to 30%.

The results of our simulations are illustrated in Supplementary Fig. 6. Supplementary Fig. 6a-d show that the main effect of crowding appears when the tracer-polymer affinity is low. As ρ increases, the volume available to the tracers diminishes, and this effect is equivalent to increasing the tracer-polymer affinity or the tracer concentration. In fact, the Pearson correlation coefficient between the polymer contacts and the tracer traffic is largely independent of the value of ρ in the case of high tracer affinity. On the other hand, it increases with increasing ρ in the case of low affinity, reaching the same value as for high tracer affinity at the same value of ϕ. We confirm this interpretation in Supplementary Fig. 6e-h by plotting the values of the mean residence time in the various cases (see Supplementary Note 3 for the definition of the mean residence time). The mean residence time is nearly 100% for the high-affinity case, independently of ρ, whereas it grows from 10-20% to 100% in the case of low affinity. Snapshots of the simulations at crowding levels of 5% and 20% are shown in Supplementary Figs. 6i and 6j, respectively.

This result is important because it shows that even if a transcription factor has low

affinity for chromatin, macromolecular crowding will enhance the effect described in the Main Text, and the traffic of the transcription factor at looping sites will increase.

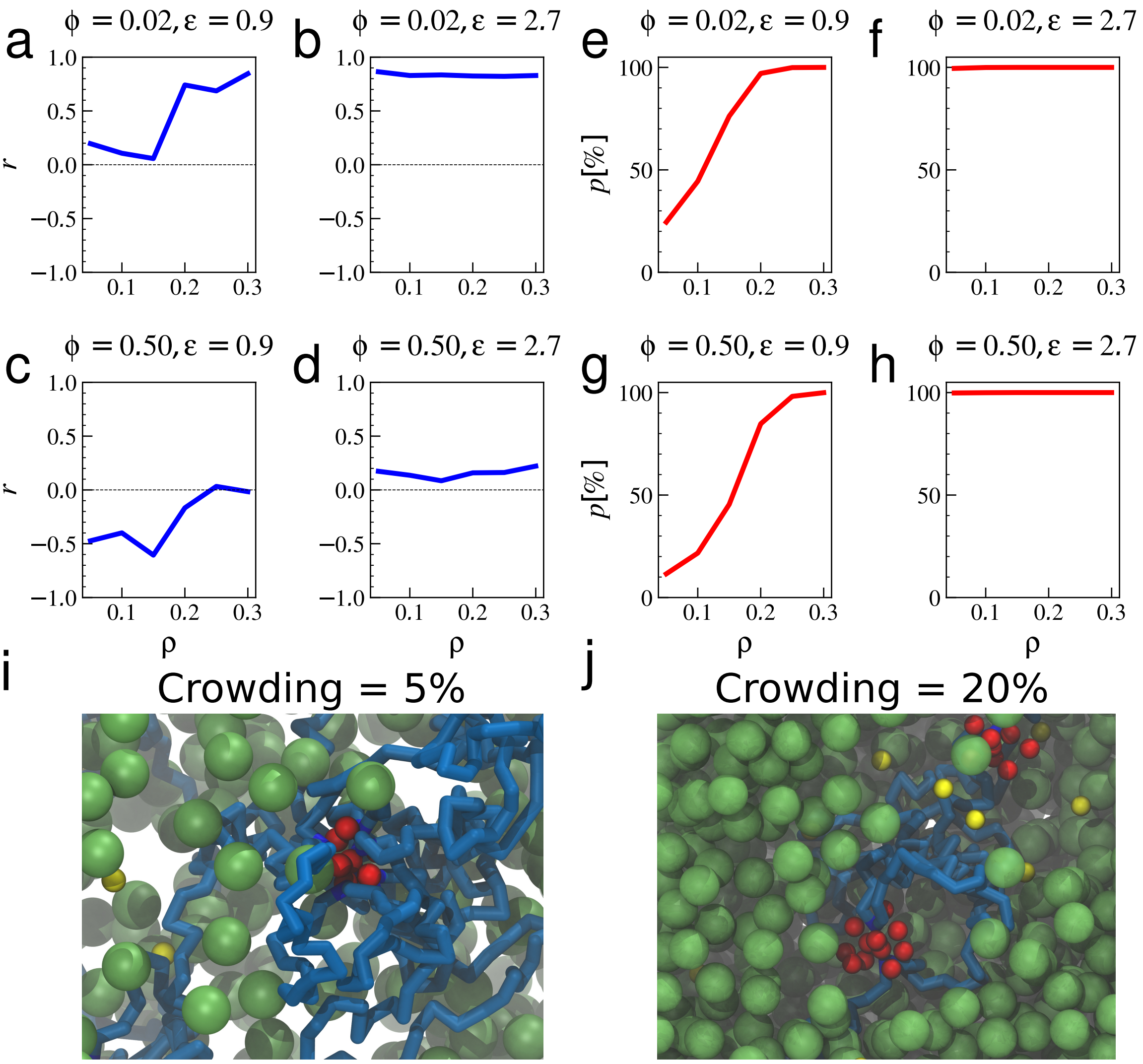

Supplementary Figure 6: Effects of crowding. (a-d) Values of the Pearson correlation coefficient between the polymer contacts and the tracer traffic as a function of the volume fraction ρ. (e-h) Percentage of time of the simulations that the tracers spend bound to the polymer, as a function of the volume fraction ρ. Visual snapshots of the simulation system at crowding level (ρ) at 5% (i) and 20% (j). The crowders are shown as green particles.

Supplementary Note 7: Convergence of the simulations

In Figure 4a of the Main Text we showed that the coverage of the tracers is different for the different simulation rounds. That is to say that the percentage of monomers that are visited at least once by the tracers is not 100%. In the case of low affinity and high compaction, the average coverage of the polymer is as low as 75%. It is natural to ask whether performing longer simulations would give a different result.

For this reason we took the 20 simulations of our simulation round with 200 tracers that had the lowest coverage, and ran the same simulations ten times longer, *i.e.* with a total of 10^9 time steps. The results of this study is illustrated in Supplementary Fig. 7.

Supplementary Fig. 7a shows that the value of the coverage in the long simulation round increases from 75% up to about 93%. To see whether this was a sign of the fact that the previous simulation had not reached convergence, we plotted the value of the Kullback-Leibler divergence between the polymer contacts and the tracer traffic as a function of time (Supplementary Fig. 7b). The KL divergence decreases monotonically and after 10^8 time steps it has already reached a plateau, indicating that the simulations had reached convergence even in the previous simulation round. We further asked whether performing significantly longer simulations would affect the tracer traffic on the polymer. In Supplementary Fig. 7c we show the tracer traffic in the previous simulation round (upper panel) and in the current one (lower panel). There is no significant difference between the traffic (the Pearson correlation coefficient between the two is 98%). The increased value of the coverage that we showed in Supplementary Fig. 7a is due to the fact that for some of the monomers the number of visits changed from zero to very few in the longer simulation run.

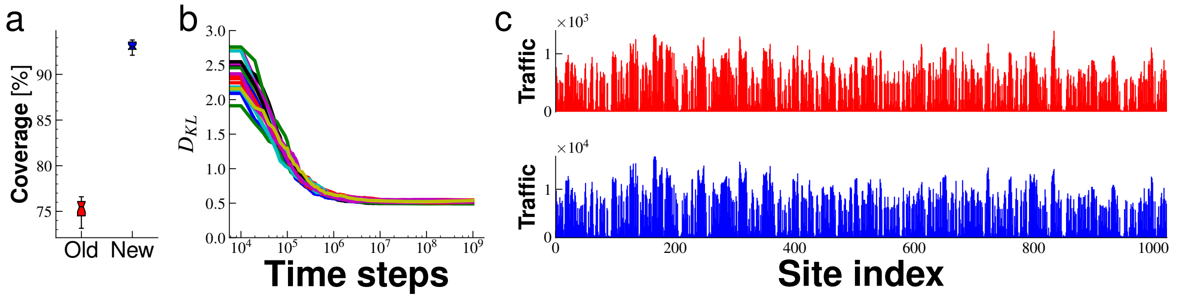

Supplementary Figure 7: Results of long simulation rounds. (a) Values of the coverage of the polymer in the previous simulation round (with 10^8 time steps) and the current simulation round (with 10^9 time steps). (b) Value of the Kullback-Leibler divergence as a function of time for each of the individual 20 simulation in our study. (c) Upper panel: traffic of the tracers in the old simulation round; lower panel: traffic of the tracers in the new simulation round.

In summary, we can be confident that the simulations that we performed have reached

convergence. Low values of the coverage indicate that some of the monomers are visited much less frequently than others.

Supplementary References

[1] Erica Uehara and Tetsuo Deguchi. Exponents of intrachain correlation for self-avoiding walks and knotted self-avoiding polygons. *Journal of Physics A: Mathematical and Theoretical*, 46(34):345001, 2013.

[2] Hong Qian, Michael P Sheetz, and Elliot L Elson. Single particle tracking. analysis of diffusion and flow in two-dimensional systems. *Biophysical journal*, 60(4):910–921, 1991.

[3] Chris A Brackley, Mike E Cates, and Davide Marenduzzo. Facilitated diffusion on mobile DNA: configurational traps and sequence heterogeneity. *Physical Review Letters*, 109(16):168103, 2012.

[4] Horng D Ou, Sébastien Phan, Thomas J Deerinck, Andrea Thor, Mark H Ellisman, and Clodagh C Oshea. ChromEMT: Visualizing 3D chromatin structure and compaction in interphase and mitotic cells. *Science*, 357(6349):eaag0025, 2017.
