# Supplementary material for: Theoretical principles of transcription factor traffic on folded chromatin
Source: Nat Commun. 2018 Apr 30;9:1740. doi: 10.1038/s41467-018-04130-x (PMC5928121; doi:10.1038/s41467-018-04130-x)
Supplement: Supplementary file 3 — Description of Additional Supplementary Files [file 41467_2018_4130_MOESM3_ESM.pdf]

Description of Additional Supplementary Files

File Name: Supplementary Movie 1
Description: Simulation of a polymer with low compaction ($\phi = 0.02$) and high tracer-polymer affinity ($\varepsilon = 2.7 k_B T$). The tracers are the yellow particles.

File Name: Supplementary Movie 2
Description: Simulation of a polymer with high compaction ($\phi = 0.50$) and high tracer-polymer affinity ($\varepsilon = 0.9 k_B T$). The tracers are the yellow particles.
